# Supplementary material for: Wnt regulates amino acid transporter Slc7a5 and so constrains the integrated stress response in mouse embryos
Source: EMBO Rep. 2019 Dec 2;21(1):e48469. doi: 10.15252/embr.201948469 (PMC6944906; doi:10.15252/embr.201948469)
Supplement: Supplementary file 1 — Appendix [file EMBR-21-e48469-s001.pdf]

Appendix Figures and Tables

Contents

Page 2
Appendix Figure S1 Validation of specificity of *Slc7a5* mRNA probe

Page 3
Appendix Figure S2 pS6 detection comparing antibodies against p-Ser 235/236 and pSer 240-244

Page 4
Appendix Figure S3 Transcription and protein levels of Slc7a5 partner Slc3a2/CD98 are not reduced in *Slc7a5*-null embryos

Page 5
Appendix Figure S4 Notch signalling is unaffected in *Slc7a5*-null embryos

Page 6
Appendix Figure S5 Expression of genes involved in the thyroid hormone response were unchanged in *Slc7a5*-null embryos and thyroid hormone receptors mRNA levels rise after E11.5 in wildtype embryos

Page 7
Appendix Figure S6 Exposure to known ER stressors Tunicamycin and Thapsigargin induces localised expression of *Trib3* and *Chac1*

Page 8
Appendix Figure S7 *Chac1* and *Trib3* transcripts are just detectable in E8.5 wildtype embryos

Page 9
Appendix Table S1 Sequence of the primers used to generate in situ hybridization probes specific for the murine *Slc7a5, Slc3a2, Chac1* and *Trib3*

Page 10
Appendix Table S2 Sequence of the primers used for qPCR

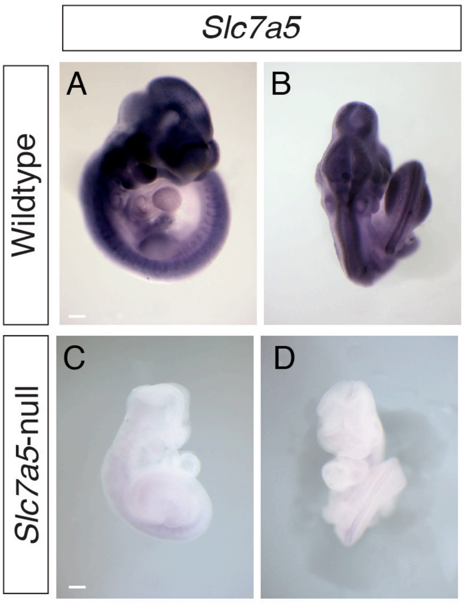

Appendix Figure S1 Validation of specificity of *Slc7a5* mRNA probe
In situ hybridisation for *Slc7a5* mRNA in (A, B) wildtype and (C, D) *Slc7a5*-null embryos. Two whole litters including n=3 mutant embryos were assessed for *Slc7a5* expression. All embryos in each litter were processed in parallel and the BCIP/NBT reaction stopped at the same time for all embryos: while strong expression of *Slc7a5* was detected in littermates, all *Slc7a5*-null embryos lacked transcripts, consistent with RNA -seq data comparing wildtype and *Slc7a5*-null embryos. Scale bar 200 µm.

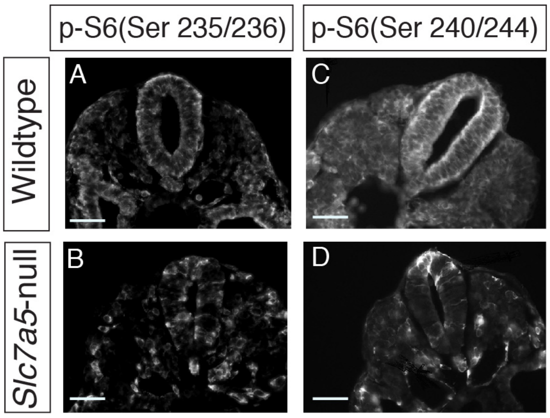

Appendix Figure S2 pS6 detection comparing antibodies against p-Ser 235/236 and pSer 240-244
The patterns of pS6 detection with an antibody recognising p-Ser 235/236 in (A) wildtype and (B) *Slc7a5*-null embryo neural tube (n=5, 18 sections and n=8, 24 sections, respectively, as in Figure 4) were found to be similar to those detected with an antibody recognising pSer 240-244 (a site specifically phosphorylated by mTORC1) in (C) wildtype and *Slc7a5*-null (D) embryos (n=1 embryo, 11 sections; n=2 embryos, 9 and 11 sections respectively). Note both antibodies reveal an aberrant /reduced pattern of pS6 in mutant embryos. Scale bar 50µm.

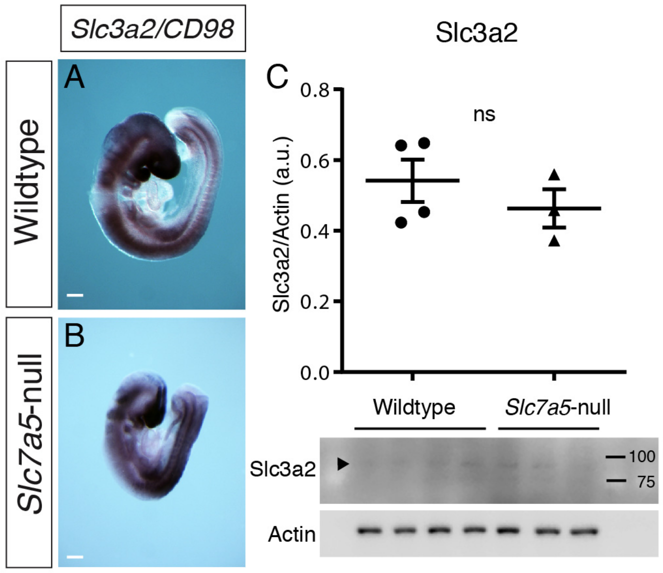

Appendix Figure S3 Transcript and protein levels of Slc7a5 partner Slc3a2/CD98 are not reduced in *Slc7a5*-null embryos

mRNA in situ hybridization was undertaken to determine whether expression of Slc7a5 partner *Slc3a2/CD98* is reduced in *Slc7a5*-null embryos. *Slc3a2* was cloned (sequence from NM_001161413.1 and probe generated for region 920-1922 - 3'end of coding sequence). *Slc3a2* transcripts were assessed in *Slc7a5*-null (n=3) and heterozygote (n=2) and wildtype (n=2) embryos at E9.5, when the phenotype is apparent. This revealed strong *Slc3a2* expression in all embryos, including (A) wildtype and (B) *Slc7a5*-null embryos, intriguingly the *Slc3a2* pattern of expression is similar to that of *Slc7a5,* Scale bars 200 µm. (C) Western blotting was used to evaluate levels of Slc3a2 protein in wildtype (n=4) and *Slc7a5*-null (n=3) embryos. Each lane contains lysate from one embryo and expression in each embryo is represented by a dot or triangle, actin is the loading control and error bars indicate SEM.

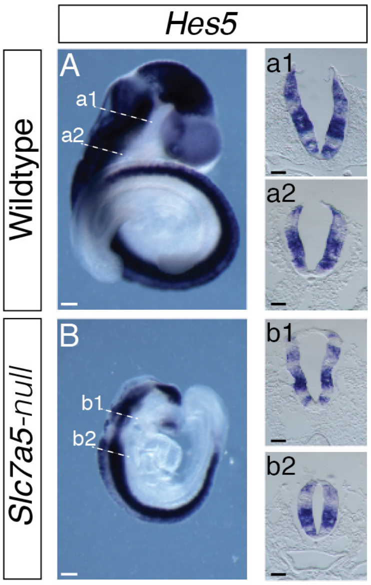

Appendix Figure S4 Notch signalling is unaffected in *Slc7a5*-null embryos
Expression of notch signalling target *Hes5* was assessed by mRNA in situ hybridization in wildtype littermate (A) and *Slc7a5*-null (B) E9.5 embryos (n=2 each). Panels (a1 – b2) show transverse sections of the dorsal neural tube at the level of the hindbrain (a1, b1) or the spinal cord (a2, b2). Scale bars 200 µm, except for sections 50 µm.

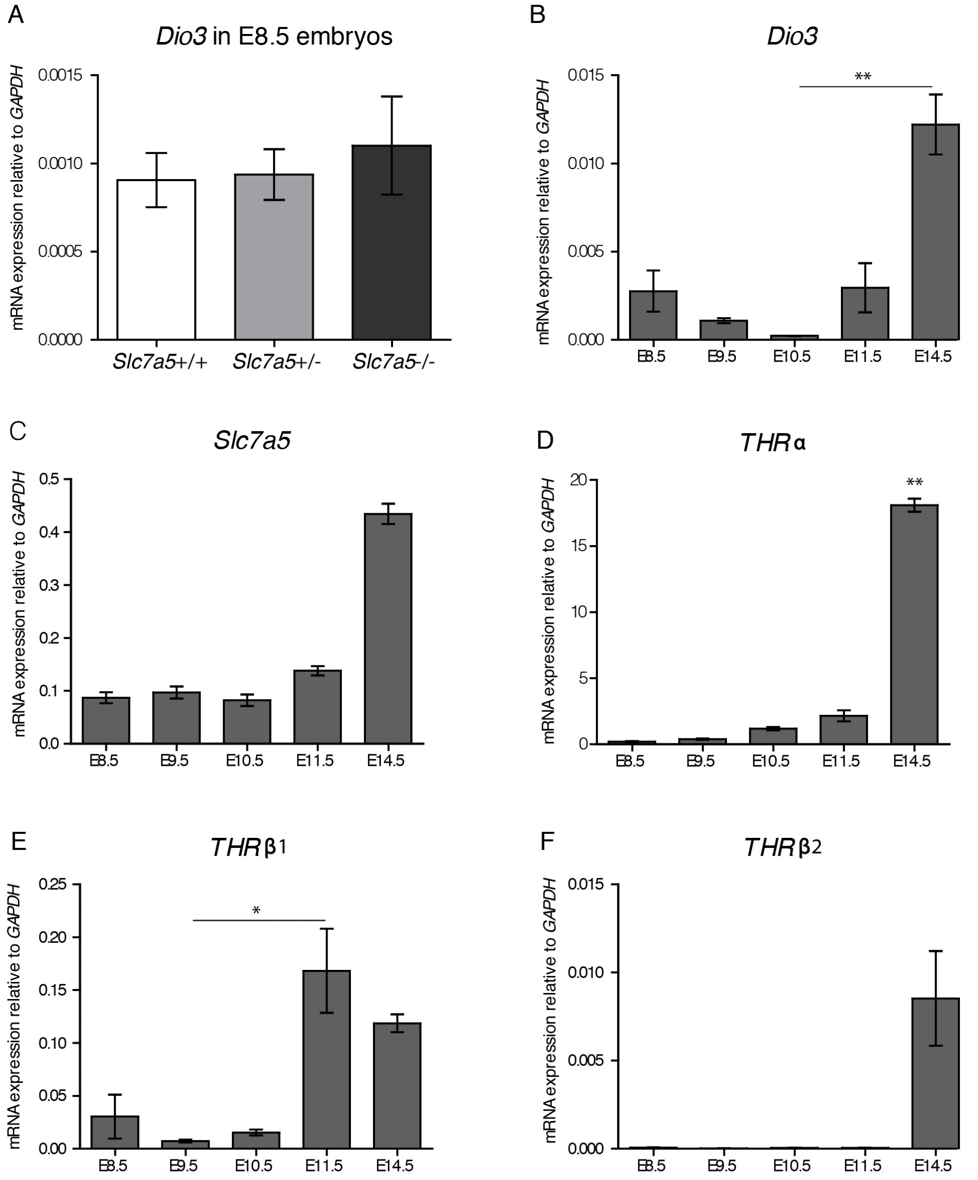

Appendix Figure S5 Expression of genes involved in the thyroid hormone response were unchanged in *Slc7a5*-null embryos and thyroid hormone receptor mRNA levels rise after E11.5 in wildtype embryos

The expression of enzyme type III iodothyronine deiodinase (*Dio3*) is T3 responsive (Bianco and Kim 2006) but was not significantly changed in RNAseq data. (A) This was confirmed using qPCR on wildtype and *Slc7a5*-deficient E8.5 embryos (n=5).

(B-F) qPCR analysis was performed on wildtype embryos at various developmental stages (n=3, except for E9.5 (n=4) using primers specific for (B) *Dio3*, (C) *Slc7a5* or (D, E, F) thyroid hormone receptors *THR*.

Data information: Error bars indicate SEM. (B-F) A one-way Anova test was performed for statistical analysis *p<0.05, **p<0.01. Actual p values for all comparisons are in original source data.

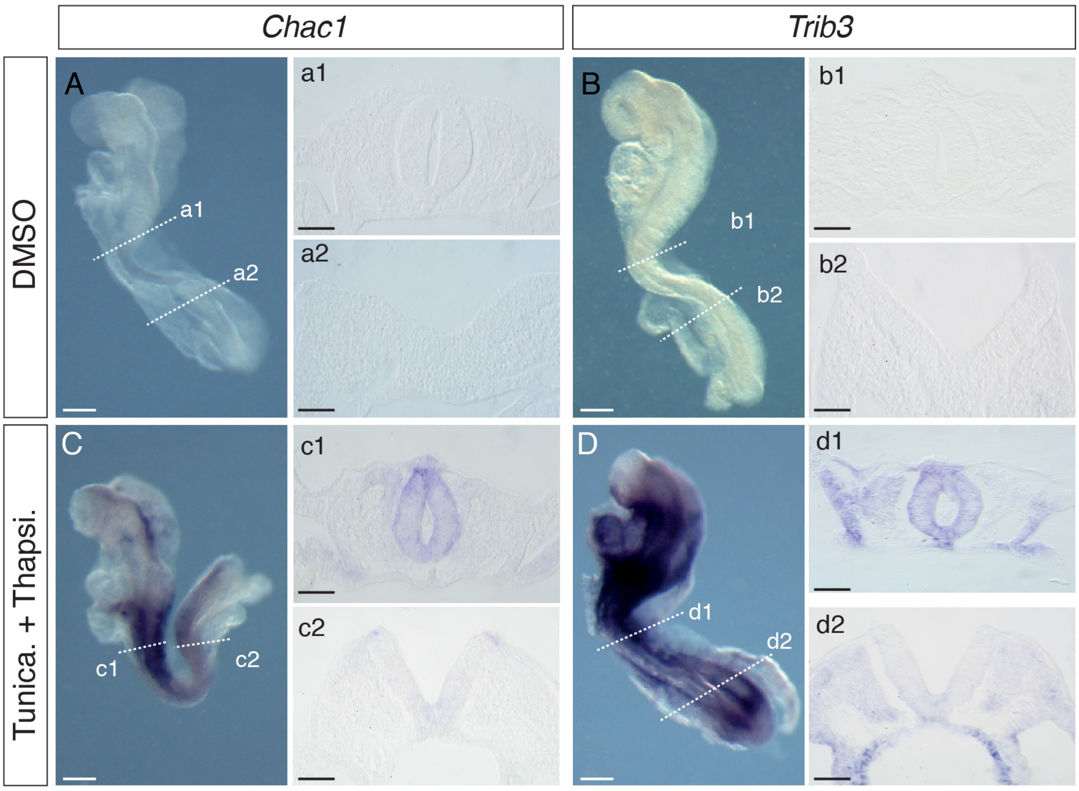

Appendix Figure S6 Exposure to known ER stressors Tunicamycin and Thapsigargin induces localised expression of *Trib3* and *Chac1*

Tunicamycin and Thapsigargin act respectively by inhibition of protein glycosylation or reduction of activity of calcium-dependent ER chaperones (Kuo & Lampen, 1974, Thastrup, Cullen et al., 1990). Exposure to either of these drugs leads to accumulation of unfolded proteins in the ER, which provokes ER stress (Oslowski & Urano 2011). Whole E8.5 embryos were suspended in hanging drop culture for 6 hours in the presence of control carrier DMSO or Tunicamycin and Thapsigargin. All embryos were harvested and processed in parallel for mRNA in situ hybridization (n=3 embryos in each condition and for each gene assessed). *Chac1* and *Trib3* transcripts were undetectable in control DMSO-only treated embryos (Figures A,B), but strongly upregulated following Tunicamycin and Thapsigargin treatment, particularly in the forming neural tube (Figures C-c2, D-d2). These data directly link increased expression of *Chac1* and *Trib3* to an ER stress response in the developing mouse embryo and show that at this early stage the developing neural tube is particularly vulnerable to such stress. Scale bars 200 µm, except for sections 100 µm.

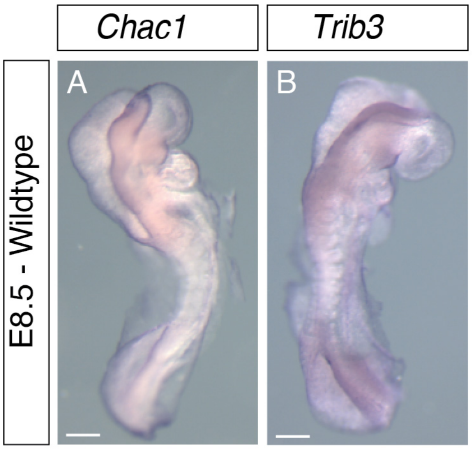

Appendix Figure S7 *Chac1* and *Trib3* transcripts are just detectable in E8.5 wildtype embryos
(A) *Chac1* and (B) *Trib3* expression pattern in wildtype E8.5 embryos after >6 days in situ hybridisation reaction (n=4 for each gene). Scale bars 200 µm.

Appendix Table S1 Sequence of the primers used to generate in situ hybridization probes specific for the murine *Slc7a5, Slc3a2, Chac1* and *Trib3*

Gene name	FASTA sequence	Forward primer	Reverse primer	product length
Slc7a5/Lat1	NM_011404.3	GCAATATCACGCTGCTCAAC	CCAAGTGGTAGTTCCCGAAG	831
Chac1	NM_026929.4	CAAGCCCTGTGGATTTTCGG	GCGGGGCATAAGACACAAAG	716
Trib3	NM_175093.2	CTTTAGCAGCGGAAGAGGCT	CTGAAGACAAAGCGACGCAG	714
Slc3a2	NM_001161413.1	GGGCTCCCAGGAAGATTTTA	TCAGGTTTTCCAGCTTCAGG	1002

Appendix Table S2 Sequence of the primers used for qPCR

Gene name	FASTA sequence	Forward primer	Reverse primer	product length
Aldh1L2	NM_153543.2	TAACACCTACAACAAGACAGAC	GTATTCATTCAGGGCCTCCTCA	96
ATF4	NM_009716.3	AGCAAAACAAGACAGCAGCC	ACTCTCTTCTTCCCCCTTGC	193
β-Actin	NM_007393.3	ATGCTCCCCGGGCTGTAT	CATAGGAGTCCTTCTGACCCATTC	87
CD98	NM_008577.4	GAGGACAGGCTTTTGATTGC	ATTCAGTACGCTCCCCAGTG	136
Chac1	NM_026929.4	AGTGTGGAAGCCGGACTTTG	CACTCGGCCAGGCATCTTGT	121
CHOP	NM_007837.4	CCACCACACCTGAAAGCAGAA	GGTGCCCCCAATTTCATCT	150
Dio3	NM_172119.2	TCAGACGACAACCGTCTGTG	AAAATTGAGCACCAACGGGC	189
Fanca	NM_016925.3	CGGGCAGAGTCAAAAAGCAA	AGCAGAGCGGATGAAGGAAG	212
GAPDH	NM_001289726.1	TGTGTCCGTCGTGGATCTGA	CCTGCTTCACCACCTTCTTGA	77
Klhdc4	NM_145605.2	CGGAAGGAGGAGGAAGACCT	TCACCTCCGAAAAGGATCAGC	164
Lefty2	NM_177099.3	GGACCTGGAGCGCACAC	GGGTCACAATTGCCTTGAGC	133
Nodal	NM_013611.4	CCATGCCTACATCCAGAGCCTGC	TGGTGTTCCAGGAGGACCCTGCC	132
Pck2	NM_028994.2	CGGCTGGAGTTCGAGACTTT	GGGCCAGCCAGCAGTTCTTA	165
Slc7a5	NM_011404.3	CTGGTCTTCGCCACCTACTT	GCCTTTACGCTGTAGCAGTTC	128
Spire2	NM_172287.2	TGGAGCCCGAGCCTACAAC	CTCTTCTGGACCCACGTAGC	227
THRα	NM_178060.3	CCTGGACAAAGACGAGCAGT	GCACTGATTCCGGGTGATCT	184
THRβ1	NM_001113417.1	GGACAAGCACCCATCGTGAAT	CTCTGGTAATTGCTGGTGTGAT	97
THRβ2	NM_009380.3	CCTGTAGTTACCCTGGAAACCTG	GGCTTTGTCCCCACACACTA	200
Trib3	NM_175093.2	TCTCCTCCGCAAGGAACCT	TCTCAACCAGGGATGCAAGAG	67
