# Supplementary material for: Wnt regulates amino acid transporter Slc7a5 and so constrains the integrated stress response in mouse embryos
Source: EMBO Rep. 2019 Dec 2;21(1):e48469. doi: 10.15252/embr.201948469 (PMC6944906; doi:10.15252/embr.201948469)
Supplement: Supplementary file 2 — Expanded View Figures PDF [file EMBR-21-e48469-s002.pdf]

Expanded View Figures

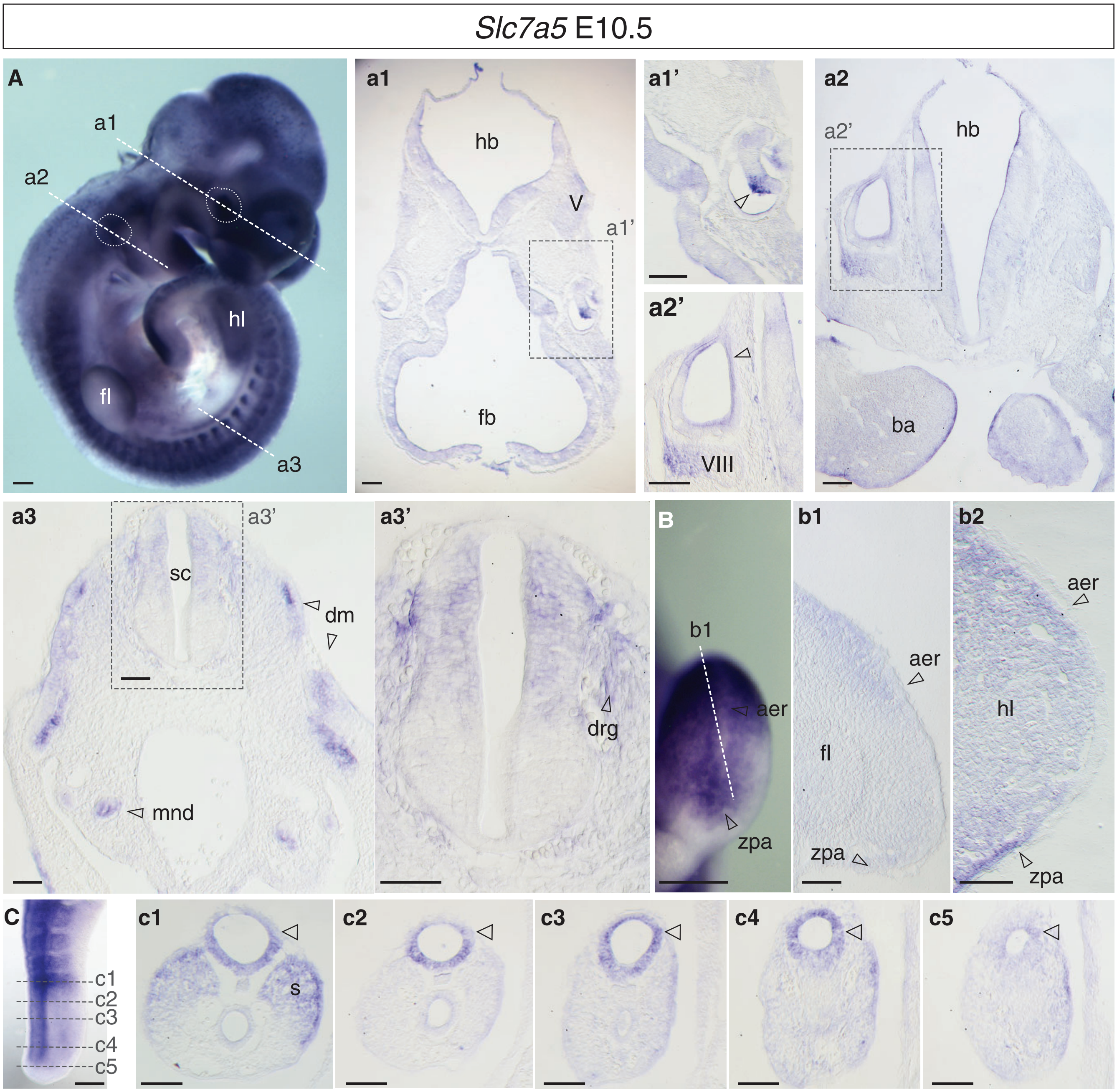

Figure EV1. *Slc7a5* mRNA expression pattern in E10.5 mouse embryo.

A E10.5 whole embryo side view and TSs (white dashed lines) showing expression in: (a1) forebrain (fb) and (a1′) optic vesicle, including regions of retina (open arrowhead) and lens; (a1, a2) hindbrain (hb), trigeminal ganglion (V) and (a2′) otic vesicle and vestibule–cochlear ganglion (VIII) and first branchial arch (ba); (a3, a3′) dorsal spinal cord (sc), dorsal root ganglia (drg), dermamyotome (dm), mesonephric duct (mnd).

B Forelimb bud (fl) (b1) and hindlimb bud (hl) (b2) in mesenchyme below the apical ectoderm ridge (aer) and in the zone of polarising activity (zpa).

C Forming somite (s) and secondary neural tube (white arrowhead).

Data information: Images representative of $n = 4$ embryos, scale bars 200 μm except for sections 100 μm.

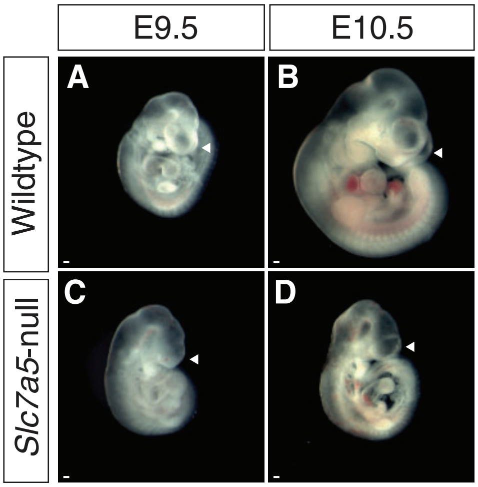

Figure EV2. A small subset of *Slc7a5*-null embryos exhibits a "flat-top-like" phenotype.

A–D Wild-type littermate (A and B) and *Slc7a5*-null embryos (C and D) were imaged at E9.5 (A and C) or E10.5 (B and D). A subset of *Slc7a5*-null embryos (~ 11%, 4/35 null embryos at E9.5 and 1/6 null embryos at E10.5) shows a "flat-top"-like phenotype with no expansion of the forebrain as described in [27] (see arrowheads). Scale bars 200 μm.

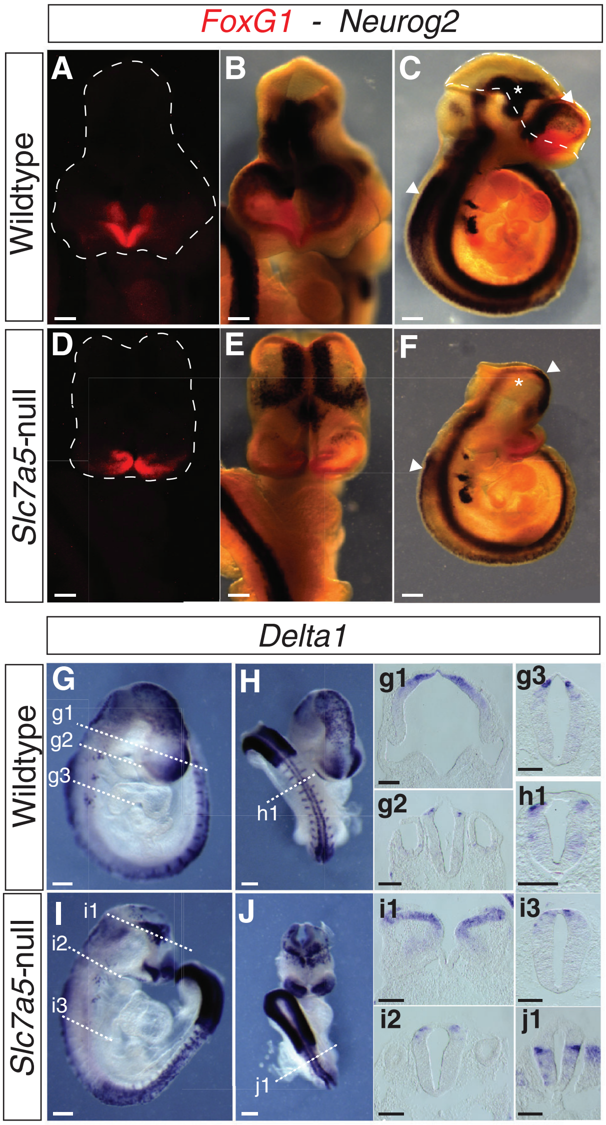

Figure EV3. *Slc7a5*-null embryos exhibit altered expression patterns of key neurogenesis genes.

A–F mRNA *in situ* hybridisation in E9.5 wild-type and *Slc7a5*-null embryos for neurogenesis marker genes. *FoxG1* and *Neurog2* double *in situ* hybridisation in E9.5 (A–C) wild-type and (D-F) *Slc7a5*-null embryos (n = 2 each for double *FoxG1*/*Neurog2*, a further n = 3 *Slc7a5*-null and n = 2 wild-type embryos were assessed for *Neurog2* expression in wholemount and sections). Images in (A and D) show frontal view of *FoxG1* mRNA detected with fast red (white dotted lines indicate shape of the head). Expression of *FoxG1* in rostral-most forebrain was detected in *Slc7a5*-null embryos with similar intensity but in a reduced domain when compared to wild type. This suggests that *Slc7a5* loss does not disrupt brain regionalisation, but leads to a reduction in the amount of tissue and/or failure of morphogenetic events underlying neural tube closure [31]. (B–F) To determine whether neurogenesis was affected in *Slc7a5*-null embryos, we assessed the expression pattern of *Neurogenin-2* (*Neurog2*), a neural progenitor marker [32]. Widespread reduction in *Neurog2* expression was apparent in whole *Slc7a5*-null embryos (E, F); some *Neurog2*-positive cell populations were represented in reduced domains (arrowheads in C, F), while others were absent (asterisk in C, F) (dotted line on the wild-type embryo (C) indicates regions of fore- and midbrain not apparent in the *Slc7a5*-null embryo).

G–J *Delta1*, a marker of newborn neurons [33], was detected in (G, H) wild type and (I, J) *Slc7a5*-null E9.5 (n = 3 each) in fore- and midbrain but showed a distorted pattern in null embryos due to neurulation failure; TSs through (g1, i1) the forebrain, (g2, i2) at the level of the otic vesicles, (g3, i3) posterior hindbrain and (h1, j1) spinal cord show reduction of the dorsally located *Delta1*-expressing cells in the *Scl7a5*-null embryos. Scale bars 200 μm, except sections 100 μm.

 © 2019 The Authors

```
                                                    GATCATTT
                                                    |-608
CGCCCTCAGACTCGTTTCCTCTGGACCAAGTTGGACTATAGGGGTGCCTCACCCCACAAC
|-600     |-590     |-580     |-570     |-560     |-550
                              Lef1/TCF motif
CCACCCCCACTTATCCTAGATAAGATATTTATCCTTCATCTCCCAGACGCACAGGATATG
|-540     |-530     |-520     |-510     |-500     |-490

CACACTGGAGGAAGCGGAGATAAACACCCGCGAGGCAGGGCCAGGATTCCGGGCAGGTGT
|-480     |-470     |-460     |-450     |-440     |-430

TGGGACTATACACTGAGCCACCCCCCTTCCGGTGCTTTCTCCCTTGACAAGTCCTCTGCG
|-420     |-410     |-400     |-390     |-380     |-370

CCGCCTGGGGCGGGAGAAGAAGCACGCCCTTACCAATCGGCCCTGACATCCTGCACAGGA
|-360     |-350     |-340     |-330     |-320     |-310

CTTGTGAGGAGGTGTGAGTCGACTCTCCACCCGCGTACAACATTCGGATCTTGTGAAACA
|-300     |-290     |-280     |-270     |-260     |-250

CGCCGCGATAAACCCATCAGTAGTCACGCCGCGTTAAACACATCAGTAGTCCTCAGTTTC
|-240     |-230     |-220     |-210     |-200     |-190
             MAX motif
AGGATCTTAACGCTCCACGTGCTCTAACAGACTTGGGGGCGGGGGCCCGCGAAGGAGGCG
|-180     |-170     |-160     |-150     |-140     |-130

GGGTCGCGCGGGCTGAGGGGGCGGGGGCCTTTGCGAGCTGAACCAACAGCAGCGGCGATG
|-120     |-110     |-100     |-90      |-80      |-70
                           Lef1/TCF motif MYC motif
GGCGGAGCCTGGAGGGCGGGTAACGGTAGAGATAAAAGGCCGCGCGGGCGGGGCGGACGC
|-60      |-50      |-40      |-30      |-20      |-10
                                   MYC motif
AGACGCACTTCTCAGTACGCGGAGAGCGGCTCGGCCGCGCGCACGCCGGGTATCCAGGCC
|TSS      |+10      |+20      |+30      |+40      |+50

GAGCCGGGAACGCCGAGAGCATGGCGGTCGCGGGCGCCAAGAGGCGCGCGGTTGCGACCC
|+60      |+70      |+80      |+90      |+110     |+100

CGGCGGCGGCGGCGGCGGAGGAGGAGCGGCAGGCGCGGGAGAAGATGTTGGAGGCGCGGC
|+110     |+120     |+130     |+140     |+150     |+160

GCGGGGACGGCGCGGACCCCGAGGGCGAAGGCGTGACCCTGCAGCGCAATATCACGCTGC
|+170     |+180     |+190     |+200     |+210     |+220

TCAACGGTGTGGCCATCATCGTGGGCACCATCATCGGCTCTGGCATCTTCGTGACGCCCA
|+230     |+240     |+250     |+260     |+270     |+280

CCGGCGTGCTCAAGGAAGCGGGGTCGCCCGGACTGTCGCTCGTGGTGTGGGCTGTGTGCG
|+290     |+300     |+310     |+320     |+330     |+340

GCGTCTTCTCCATCGTGGGCGCACTCTGCTACGCGGAGCTGGGCACCACCATCTCCAAGT
|+350     |+360     |+370     |+380     |+390     |+400

CAGGCGGCGACTACGCCTACATGCTGGAGGTCTACGGCTCGTTGCCCGCCTTCCTCAAGC
|+410     |+420     |+430     |+440     |+450     |+460

TCTGGATCGAGCTGCTCATCATTCGGCCCTCCTCGCAGTACATCGTGGCGCTGGTCTTCG
|+470     |+480     |+490     |+500     |+510     |+520

CCACCTACTTGCTCAAGCCGGTCTTCCCCACTTGTCCGGTGCCCGAGGAAGCTGCCAAGC
|+530     |+540     |+550     |+560     |+570     |+580

TCGTGGCCTGCCTCTGCGTGCGTAAGTAGGGGTCCGGGCTGGGGTGGGGCCGGGCTGGGG
|+590     |+600     |+610     |+620     |+630     |+640

CGGGGCCGGGTGGGCGCGAGCCAAGGTTCCTGCGTTTACAGCTCGGCAACACCCATCCCA
|+650     |+660     |+670     |+680     |+690     |+700
```

Figure EV4. Position and sequence of MYC and LEF/TCF binding sites in *Slc7a5* promoter region.

Analysis of the −1,075 to +352 bp sequence of the *Slc7a5* mouse gene with the MatInspector program [61] identified putative MYC binding sites at positions TSS −9/−25 and +30/+46 (with a matrix similarity comprised between 0.925 and 0.931). A further MYC/MAX binding site, similar to the MYC binding E-box motif identified in human *Slc7a5* promoter [19], was found at −TSS −154/−170. A potential LEF/TCF binding motif (sequence tagagataAAAGgccgc) at position −18/−34 from the transcription starting site. It showed a matrix similarity of 0.903 (see Dataset EV3). This motif was also found by analysing the −1,000 to +100 bp sequence of murine *Slc7a5*, using the eukaryotic promoter database (http://epd.vital-it.ch/) which is based on the JASPAR CORE 2018 vertebrate database [108]. Another motif was found at position −505 with the cut-off value set at $P \leq 0.001$. Both sites were highlighted on the murine *Slc7a5* gene sequence based on position and matrix similarity.

© 2019 The Authors

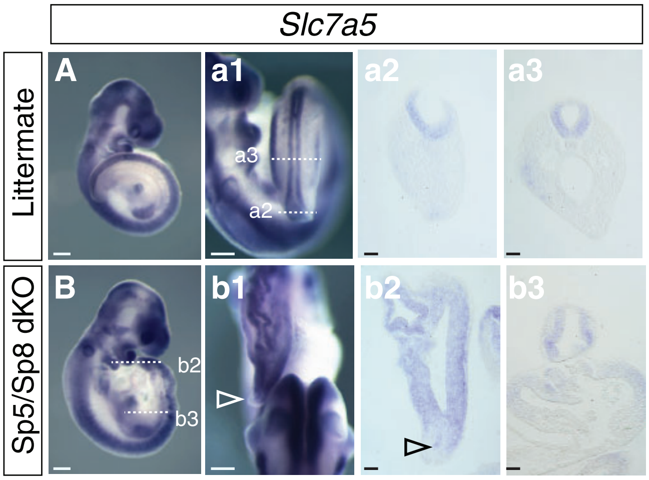

Figure EV5. Expression of *Slc7a5* in Sp5/Sp8 double-mutant embryos.

A, B *Slc7a5* is expressed to the caudal tip of the *Sp5/Sp8* double knockout embryos despite failure to form paraxial mesoderm. (A, a1) *Slc7a5* expression in littermate control, (a2, a3) in TS, and (B, b1) in *Sp5/Sp8* double knockout (dKO) embryo, (b2, b3) in TS (n = 5/5 mutant embryos and n = 3/3 littermate controls). Open arrowheads indicate posterior neural tube. Scale bars 200 µm, except sections 50 µm.

 © 2019 The Authors
