# Supplementary material for: Wnt regulates amino acid transporter Slc7a5 and so constrains the integrated stress response in mouse embryos
Source: EMBO Rep. 2019 Dec 2;21(1):e48469. doi: 10.15252/embr.201948469 (PMC6944906; doi:10.15252/embr.201948469)
Supplement: Supplementary file 6 — Source Data for Appendix [file EMBR-21-e48469-s009.zip › Appendix_Source_Data/Appendix_Figure_S3/Appendix_Figure_S3_WB_CD98.pdf]

CD98

Immunoblot performed using anti-CD98 Antibody (Santa Cruz #sc-20018) applied overnight.

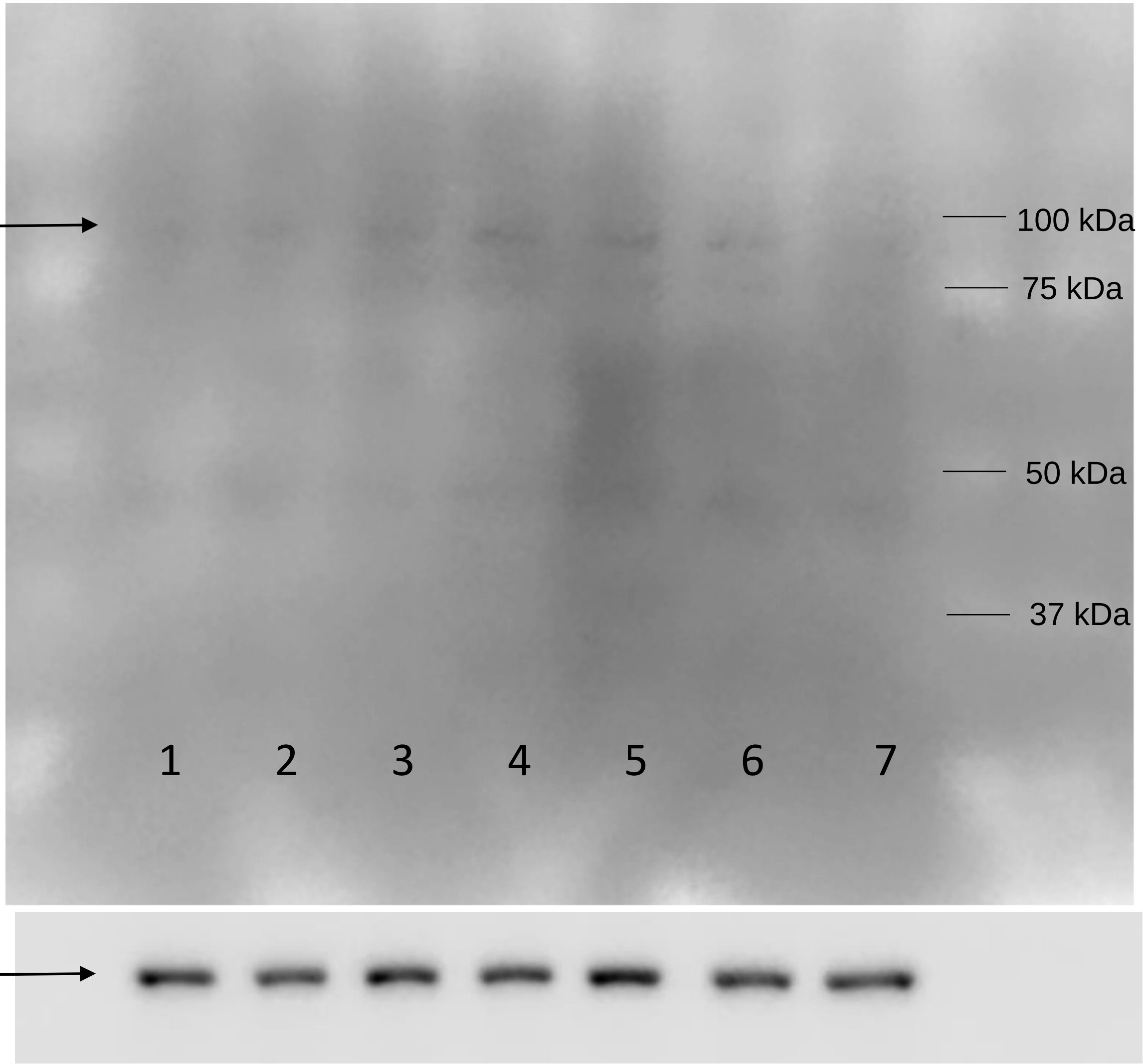

Actin (Sigma Antibody)

Lanes:

1. WT-E5L15
2. WT-E6L15
3. WT-E4L16
4. WT-E6L16
5. Mut-E8L15
6. Mut-E5L16
7. Mut-E3L17

25 µg protein loaded per lane
