# Supplementary figures and images for: Wnt regulates amino acid transporter Slc7a5 and so constrains the integrated stress response in mouse embryos
Source: EMBO Rep. 2019 Dec 2;21(1):e48469. doi: 10.15252/embr.201948469 (PMC6944906; doi:10.15252/embr.201948469)

- wt 2
- wt 5
- wt 6
- Hom 1
- Home
- Hom h
- Humf
- Het 7
- Het 8
- Het a
- Het b

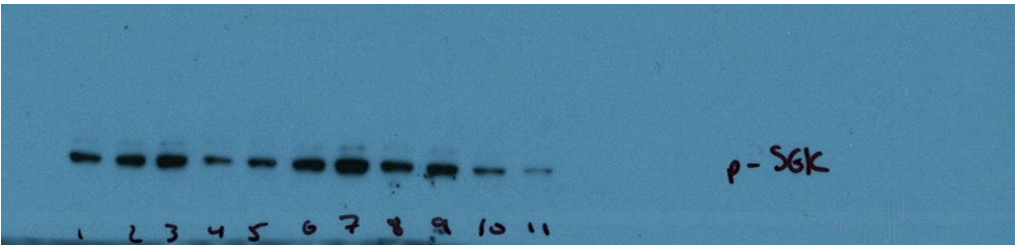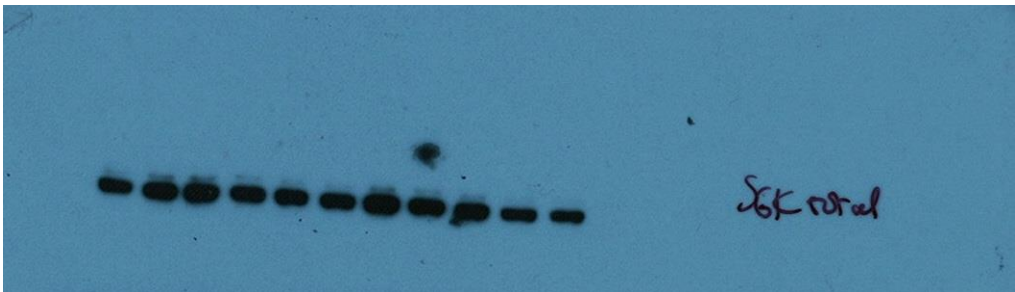

Supplement: Supplementary file 9 — Source Data for Figure 4 [file EMBR-21-e48469-s007.zip › Fig4_Source_Data/Figure_4_WB_pS6K_and_S6K.pdf]

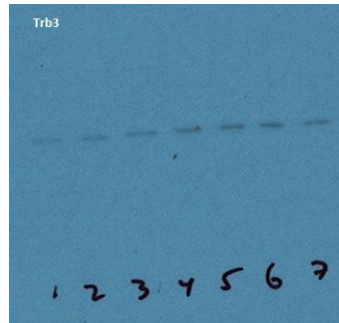

Trib3 Short exposure time

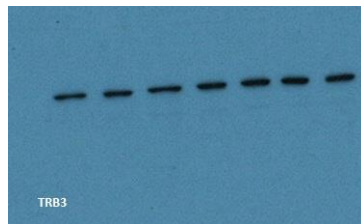

Trib3 Long exposure time

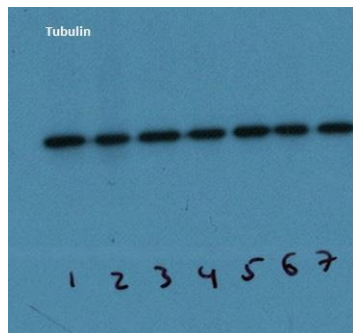

Tubulin

1. wt 2  
2. wt 5  
3. wt 6  
4. Hom1  
5. Homc  
6. Homh  
7. Homf

Supplement: Supplementary file 10 — Source Data for Figure 6 [file EMBR-21-e48469-s008.zip › Fig6_Source_Data/Figure_6_WBTrib3.pdf]
