# Supplementary material for: Wnt regulates amino acid transporter Slc7a5 and so constrains the integrated stress response in mouse embryos
Source: EMBO Rep. 2019 Dec 2;21(1):e48469. doi: 10.15252/embr.201948469 (PMC6944906; doi:10.15252/embr.201948469)
Supplement: Supplementary file 10 — Source Data for Figure 6 [file EMBR-21-e48469-s008.zip › Fig6_Source_Data/Figure_6_WB_peIF2a_and_eIF2a.pdf]

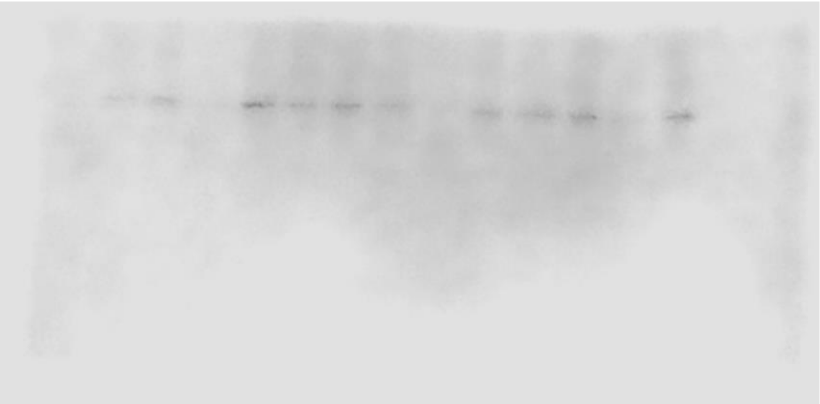

Phospho-eIF2alpha

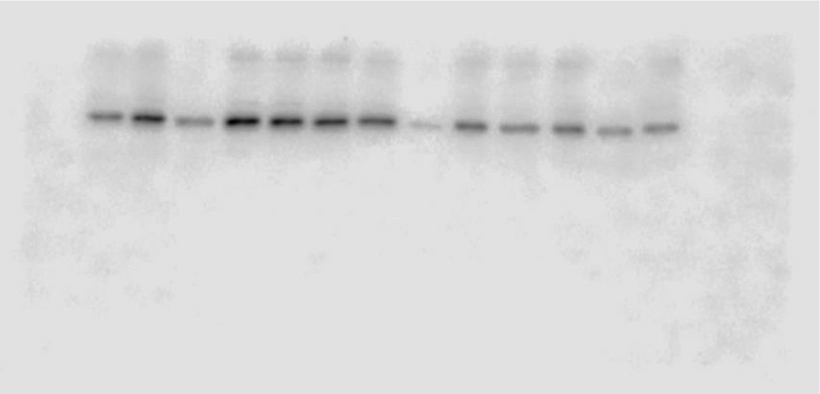

total-eIF2alpha

Embryo #	Lane
WT_L15E5	1
WT_L15E6	2
WT_L16E1	3
WT_L16E4	4
WT_L16E6	5
Mut_L15E8	6
Mut_L16E5	7
Mut_L17E2	8
Mut_L17E3	9
Mut_L17E6	10
Het_L15E3	11
Het_L16E2	12
Het_L17E4	13
